# Nanoresonator Enhancement of Majorana-Fermion-Induced Slow Light in Superconducting Iron Chains

**DOI:** 10.3390/mi12121435

**Published:** 2021-11-23

**Authors:** Huajun Chen

**Affiliations:** School of Science, Anhui University of Science and Technology, Huainan 232001, China; hjchen@aust.edu.cn

**Keywords:** Majorana fermions, nanomechanical resonator, quantum dot, slow light

## Abstract

We theoretically investigate Fano resonance in the absorption spectrum of a quantum dot (QD) based on a hybrid QD-nanomechanical resonator (QD–NR) system mediated by Majorana fermions (MFs) in superconducting iron (Fe) chains. The absorption spectra exhibit a series of asymmetric Fano line shapes, which are accompanied by the rapid normal phase dispersion and induce the optical propagation properties such as the slow light effect under suitable parametric regimes. The results indicated that the slow light induced by MFs can be obtained under different coupling regimes and different detuning regimes. Moreover, we also investigated the role of the NR, and the NR behaving as a phonon cavity enhances the slow light effect.

## 1. Introduction

Majorana fermions (MFs) have witnessed significant progress in the past few decades in condensed matter systems due to their potential applications in decoherence-free quantum computation and quantum information processing [1] because they obey non-Abelian statistics. Although proposed originally as a model for neutrinos, MFs have recently been predicted to occur as quasi-particle bound states in engineered solid state systems [2], such as semiconducting nanowire/superconductor (SNW/SC) hybrid structure [3,4,5,6], ferromagnetic atomic chains on a superconductor [7], iron-based superconductor hybrid devices [8], topological superconductors [9,10], and topological insulator structures [11]. To observe Majorana-like signatures in the hybrid solid-state systems, a few significant and representative experimental schemes have been proposed, such as the zero-bias peaks (ZBPs) in tunneling spectroscopy [3,4,5,6,7], the Josephson effect [12], Coulomb blockade spectroscopy [9], and the spin-resolved measurements [13].

On the other hand, due to the significant progress in modern nanoscience and nanotechnology, artificial atoms, i.e., quantum dots (QDs) [14], indicate the ideal intermediary to detect and investigate MFs both theoretically [15,16,17,18,19] and experimentally [20]. We found that QDs are always treated as only a resonant level [15,16,17,18,19] in the detection of MFs with QDs in the electrical domain. Different from the previous schemes for detecting MFs, we presented an optical scheme for probing MFs with QD treated as a two-level system (TLS) and driven by the optical pump–probe technology [21,22], which may provide a potential supplement for detecting MFs. In order to investigate MF-induced coherent optical propagation, such as the slow light effect, we designed a hybrid QD–SNW/SC ring device [23,24], and to reach the enhanced coherent optical spectra, we considered putting the QD into a nanomechanical resonator (NR) system [21,22]. However, the NR enhancement of the coherent optical propagation, such as the fast and slow light effects [25,26,27] mediated by MFs, have not yet been explored to the best of our knowledge.

**Figure 1:**
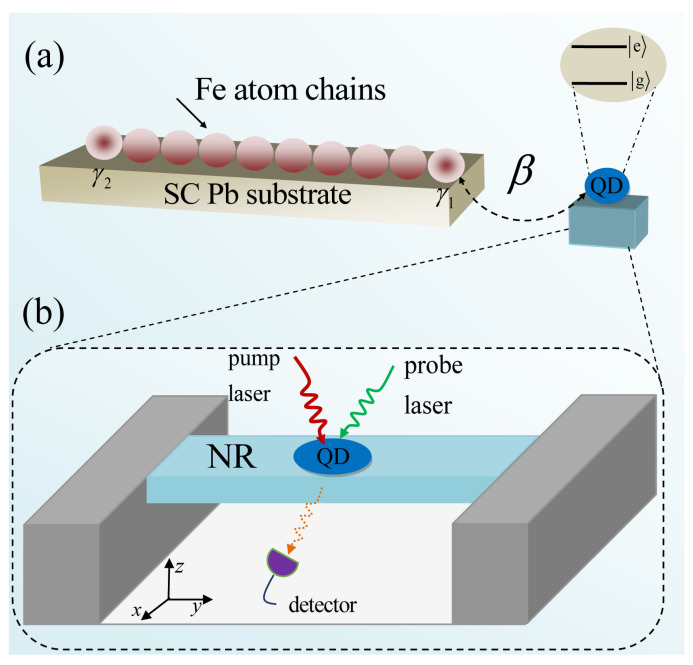
(**a**) The schematic of the hybrid device, where a two-level QD is coupled to a nearby MF in the iron chains on the superconducting Pb surface. (**b**) The QD driven by two-tone fields is embedded in an NR.

In this paper, motivated by the above-mentioned optical schemes for detecting MFs, we investigated the Fano resonance and slow light in the hybrid system discussed in [22], where a two-level QD was implanted in an NR to probe MFs in ferromagnetic atomic chains on a superconductor. Previous works for detecting MFs by optical means have shown that both the linear [22] and nonlinear [21] optical spectra are enhanced significantly when the NR is introduced. In the present paper, we first investigated the absorption spectra of the QD in the hybrid QD–NR system and the iron chains on the superconducting Pb surface, as shown in Figure 1, under different system parameters including the QD–MF coupling strength β, the QD–NR coupling strength *g*, the Majorana–pump field detuning $\Delta_{M}$, and the exciton–pump field detuning $\Delta_{c}$. Especially, the absorption spectra display a series of asymmetric Fano line shapes when considering QD–MF coupling, which can be illustrated using the interference effect with the dressed state theory. Due to Fano resonances being characterized by a rapid, steeper dispersion, the group velocity index $n_{g}$ of light pulses can be accelerated and decreased significantly, which correspond to the negative and positive dispersion, respectively, and then reach the fast and slow light effects. Second, we investigated the slow light effect by numerically calculating the group delay of the probe field around the transparency window accompanied by the steep phase dispersion, and we found that a tunable and controllable slow light propagation can be achieved by manipulating the parametric regimes. Moreover, the slow light effect was enhanced significantly compared with previous work [24] because, here, the NR was introduced in the present system, which acts as a phonon cavity, enhancing the slow light effect.

## 2. Model and Theory

### 2.1. The Hamiltonian of the System

The schematic setup studied in this work is shown in Figure 1, where iron (Fe) chains are overlaid on the superconducting Pb(110) surface [7], and we employed a two-level QD driven by two-tone fields to investigate the coherent optical properties mediated by MFs [23]. To obtain the enhanced coherent optical properties, we considered that the two-level QD is implanted in an NR system to make up a hybrid QD–NR system [22]. Here, based on the hybrid system as shown in Figure 1, we investigated MF-induced coherent optical propagation. The QD was treated as a TLS consisting of the ground state $|g\rangle$ and the single exciton state $|e\rangle$ in the hybrid QD–NR system, and $H_{QD}=\hbar \omega_{ex}S^{z}$ with the exciton frequency $\omega_{ex}$ is the Hamiltonian of the QD, where $S^{z}$ and $S^{\pm }$ are the pseudospin operators with commutation relations $\left[S^{z},S^{\pm }\right]=\pm S^{\pm }$ and $\left[S^{+},S^{-}\right]=2S^{z}$. For the NR [28,29], which can be characterized by a harmonic oscillator with Hamiltonian $H_{NR}=\hbar \omega_{m}b^{+}b$ (where *b* and $\omega_{m}$ are the annihilation operator and frequency of the phonon mode), due to the thickness of the beam being much smaller than its width, and then, the lowest-energy resonance corresponding to the fundamental flexural mode will constitute the phonon mode. In the hybrid QD–NR system [28], the flexion induces extensions and compressions, and then, the longitudinal strain will modify the energy of the electronic states of QDs via deformation potential coupling with the coupled Hamiltonian $H_{cou}=\hbar \omega_{m}\beta S^{z}\left(b^{+}+b\right)$ with the coupling strength β [28]. As a result, we obtain the Hamiltonian of the hybrid QD–NR system as:

(1) $$H_{QD--NR}=\hbar \omega_{ex}S^{z}+\hbar \omega_{m}b^{+}b+\hbar \omega_{m}\beta S^{z}\left(b^{+}+b\right).$$

For the QD–MF coupling, due to each MF being its own antiparticle, then an operator γ with $\gamma^{†}=\gamma$ and $\gamma^{2}=1$ is introduced to describe MFs. We obtain that the Hamiltonian of the QD couples to the nearby MF $\gamma_{1}$ as follows [15,16,17,18,19,21,22,23,24]:

(2) $$H_{QD--MF}=i\epsilon_{M}\gamma_{1}\gamma_{2}/2+i\hbar g\left(S^{-}-S^{+}\right)\gamma_{1},$$

where the first term in Equation (2) is the interaction of the two MFs in Fe chains on the superconducting Pb. $\epsilon_{M}=\hbar \omega_{M}∼e^{-l/\xi }$ is the coupling energy with *l* being the length of the Fe chains and ξ the superconducting Pb coherent length with Majorana frequency $\omega_{M}$. If the length *l* is large enough, we find the coupling energy $\epsilon_{M}∼e^{-l/\xi }∼0$. Therefore, we need to discuss the two cases, i.e., $\epsilon_{M}\neq 0$, termed coupled Majorana edge states, and $\epsilon_{M}=0$, termed uncoupled Majorana edge states. The second one in Equation (2) gives the near by MFs $\gamma_{1}$ coupled to the QD with the coupling strength β, which relate to the distance of the QD and superconducting device. For simplicity, we introduce the regular fermion creation and annihilation operators $f^{†}$ and *f* with the anti-commutative relation $\left\{f,\;f^{†}\right\}=1$; thus, according to the relation of $\gamma_{1}=f^{†}+f$ and $\gamma_{2}$$=i(f^{†}-f)$, Majorana operator γ can be transformed into the regular fermion operator *f*. Then, the second term in Equation (2) reduces to $i\hbar g(S^{-}f^{†}-S^{+}f)$ by neglecting the non-conservation terms of energy $i\hbar g(S^{-}f-S^{+}f^{+})$ based on the rotating wave approximation [30]. Therefore, Equation (2) can be reduced to:

(3) $$H_{QD--MF}=\hbar \Delta_{M}\left(f^{†}f-1/2\right)+i\hbar g\left(S^{-}f^{†}-S^{+}f\right).$$

When the hybrid QD–NR system is driven by two-tone fields [31], the Hamiltonian of QD coupled to the two fields is given by [32]:

(4) $$H_{Dri-QD}=-\mu \varepsilon_{p}\left(S^{+}e^{-i\omega_{c}t}+S^{-}e^{i\omega_{c}t}\right)-\mu \varepsilon_{s}\left(S^{+}e^{-i\omega_{s}t}+S^{-}e^{i\omega_{s}t}\right),$$

where μ is the dipole moment of the exciton, $\omega_{c}$ ($\omega_{s}$) is the strong pump (weak probe) field frequency, and $\varepsilon_{c}$ and $\varepsilon_{s}$ are the slowly varying envelope of the pump field and probe field, respectively. In a rotating frame of frequency $\omega_{p}$, we obtain the total Hamiltonian of the system as:

(5) $$\begin{array}{cc} H & =\hbar \Delta_{c}S^{z}+\hbar \omega_{m}b^{+}b+\hbar \omega_{m}\beta S^{z}\left(b^{+}+b\right)+\hbar \Delta_{M}\left(f^{†}f-1/2\right)+i\hbar g\left(S^{-}f^{†}-S^{+}f\right)\\ \; & -\hbar \Omega_{c}\left(S^{+}+S^{-}\right)-\mu \varepsilon_{s}\left(S^{+}e^{-i\delta t}+S^{-}e^{i\delta t}\right), \end{array}$$

where $\Delta_{c}=\omega_{ex}-\omega_{c}$ ($\delta =\omega_{s}-\omega_{c}$, $\Delta_{M}=\omega_{M}-\omega_{c}$) is the detuning of the exciton frequency (probe field, the MF frequency) from the pump frequency. $\Omega_{c}=\mu \varepsilon_{c}/\hbar$ is the Rabi frequency of the pump field.

### 2.2. Quantum Langevin Equations

According to the Heisenberg equation of motion iℏ∂ρ/∂t=[ρ,H] ($\rho =S^{z}$, $S^{-}$, *f*, *Q*), we can obtain the Heisenberg–Langevin equations (H-LEs) of the operators with the corresponding noise and damping terms as follows:

(6) $${\dot{S}}^{z}=-\Gamma_{1}\left(S^{z}+1/2\right)-g\left(S^{-}f^{†}+S^{+}f\right)+i\Omega_{c}\left(S^{+}-S^{-}\right)+\left(i\mu \varepsilon_{s}/\hbar \right)\left(S^{+}e^{-i\delta t}-S^{-}e^{i\delta t}\right),$$

(7) $${\dot{S}}^{-}=-\left[\left(i\Delta_{c}+\omega_{m}\beta Q\right)+\Gamma_{2}\right]S^{-}+2gS^{z}f-2i\Omega_{c}S^{z}-2i\mu \varepsilon_{s}S^{z}e^{-i\delta t}/\hbar +\hat{\tau }\left(t\right),$$

(8) $$\dot{f}=-\left(i\Delta_{M}+\kappa_{M}/2\right)f+gS^{-}+\hat{\varsigma }\left(t\right),$$

(9) $$\ddot{Q}+\gamma_{m}\dot{Q}+\omega_{m}^{2}Q=-2\omega_{m}^{2}\beta S^{z}+\hat{\xi }\left(t\right),$$

where $\Gamma_{1}$ ($\Gamma_{2}$) is the exciton spontaneous emission rate (dephasing rate), $Q=b^{†}+b$ is the position operator, and $\gamma_{m}$ ($\kappa_{M}$) is the decay rate of the NR (MF). $\hat{\tau }\left(t\right)$ is the δ-correlated Langevin noise operator with zero mean and obeys the correlation function $\langle \hat{\tau }\left(t\right){\hat{\tau }}^{†}\left(t\right)\rangle ≃\delta \left(t-t^{{}^{\prime }}\right)$. $\hat{\varsigma }$ and $\hat{\xi }$ represent the Langevin force arising from the interaction between the Majorana modes and the environment [22,33].

We introduce the perturbation theory: $\rho =\rho_{0}+\delta \rho$, then $\rho_{0}$ (i.e., $S_{0}^{z}$, $S_{0}$, and $f_{0}$) means the steady parts and δρ (i.e., $\delta S^{z}$, $\delta S^{-}$, and δf) indicates the fluctuation ones. Substituting the perturbation method into Equations (6)–(9), we obtain the steady-state solutions of the variables as follows:

(10) $$\Gamma_{1}\left(w_{0}+1\right)+2g\left(S_{0}f_{0}^{*}+S_{0}^{*}f_{0}\right)=2i\Omega_{c}\left(S_{0}-S_{0}^{*}\right),$$

(11) $$\left(i\Delta_{c}^{{}^{\prime }}+\Gamma_{2}\right)S_{0}=2\left(gf_{0}-i\Omega_{c}\right),$$

(12) $$\left(i\Delta_{M}+\kappa_{M}/2\right)f_{0}=gS_{0},\;Q_{0}=-\beta w_{0},$$

which determine the steady-state population inversion ($w_{0}=2S_{0}^{z}$) of the exciton as follows:

(13) $$\Gamma_{1}\left(w_{0}+1\right)\left(\Xi_{1}\Xi_{2}+\beta^{2}w_{0}^{2}\Xi_{3}\right)+4w_{0}\Gamma_{2}\Omega_{pu}^{2}\Xi_{1}=0.$$

where $\Xi_{1}=\Delta_{M}^{2}+\kappa_{M}^{2}/4$, $\Xi_{2}=\left(\Delta_{c}^{2}+\Gamma_{2}^{2}+\omega_{m}^{2}g^{4}w_{0}^{2}-2\omega_{m}\Delta_{c}g^{2}w_{0}\right)$, $\Xi_{3}=\beta^{2}-2\omega_{m}\Delta_{M}g^{2}+2\Delta_{c}\Delta_{M}-\Gamma_{2}\kappa_{M}$, and $\Delta_{c}^{{}^{\prime }}=\Delta_{c}+\omega_{m}\beta Q_{0}$. As all the pump fields are assumed to be sufficiently strong, all the operators can be identified with their expectation values under the mean-field approximation $\langle Qc\rangle =\langle Q\rangle \langle c\rangle$ [34], and after being linearized by neglecting nonlinear terms in the fluctuations, the H-LEs for the expectation values are:

(14) $$\begin{array}{ccc} \langle \delta {\dot{S}}^{z}\rangle & = & -\Gamma_{1}\langle \delta S^{z}\rangle -g\left(S_{0}\langle \delta f^{†}\rangle +S_{0}^{*}\langle \delta f\rangle +f_{0}^{*}\langle \delta S^{-}\rangle +f_{0}\langle \delta S^{+}\rangle \right)+i\Omega_{p}\left(\langle \delta S^{+}\rangle -\langle \delta S^{-}\rangle \right)\\ & & +\frac{i\mu \varepsilon_{s}}{\hbar }\left(S_{0}^{*}e^{-i\delta t}-S_{0}e^{i\delta t}\right), \end{array}$$

(15) $$\langle \delta {\dot{S}}^{-}\rangle =-\left(i\Delta_{c}^{{}^{\prime }}+\Gamma_{2}\right)\langle \delta S^{-}\rangle -2i\Omega_{c}\langle \delta S^{-}\rangle +2g\left(f_{0}\langle \delta S^{-}\rangle +S_{0}\langle \delta f\rangle \right)-\frac{i\mu \varepsilon_{s}w_{0}e^{-i\delta t}}{\hbar },$$

(16) $$\langle \delta \dot{f}\rangle =-\left(i\Delta_{M}+\kappa_{M}/2\right)\langle \delta f\rangle +g\langle \delta S^{-}\rangle ,$$

(17) $$\langle \delta \ddot{Q}\rangle +\gamma_{m}\langle \delta \dot{Q}\rangle +\omega_{m}^{2}\langle \delta Q\rangle =-2\omega_{m}^{2}\beta \langle \delta S^{z}\rangle .$$

### 2.3. Coherent Optical Spectrum

In order to solve the equation set of the above H-LEs, we make the ansatz [32] $\langle \delta \rho \rangle =\rho_{+}e^{-i\delta t}+\rho_{-}e^{i\delta t}$. Solving the equation set and working to the lowest order in $\varepsilon_{s}$, but to all orders in $\varepsilon_{p}$, we obtain the linear optical susceptibility as $\chi_{eff}^{\left(1\right)}\left(\omega_{s}\right)=\mu S_{+}\left(\omega_{s}\right)/\varepsilon_{s}=\Sigma_{1}\chi^{\left(1\right)}\left(\omega_{s}\right)$ with $\Sigma_{1}=\mu^{2}/\left(\hbar \Gamma_{2}\right)$, and then, the dimensionless linear susceptibility $\chi^{\left(1\right)}\left(\omega_{s}\right)$ is given by:

(18) $$\chi^{\left(1\right)}\left(\omega_{pr}\right)=\frac{\left[{(\Pi_{4}^{*}+\Lambda }_{1}\Pi_{3}^{*})\Pi_{1}\Lambda_{3}-iw_{0}\Pi_{4}^{*}\right]\Gamma_{2}}{\Pi {}_{2}\Pi_{4}^{*}-\Lambda_{1}\Lambda_{2}\Pi_{1}\Pi_{3}^{*}},$$

where $\Sigma_{1}=g/\left(i\Delta_{M}+\kappa_{M}/2-i\delta \right)$, $\Sigma_{2}=g/\left(-i\Delta_{M}+\kappa_{M}/2-i\delta \right)$, $\eta =2\beta \omega_{m}^{2}/\left(\delta^{2}+i\delta \gamma_{m}-\omega_{m}^{2}\right)$, $\Lambda_{1}=\left[i\Omega_{c}-g\left(f_{0}+S_{0}\Sigma_{2}^{*}\right)\right]/\left(\Gamma_{1}-i\delta \right)$, $\Lambda_{2}=\left[-i\Omega_{c}-g\left(f_{0}^{*}+S_{0}^{*}\Sigma_{1}\right)\right]/\left(\Gamma_{1}-i\delta \right)$, $\Lambda_{3}=iS_{0}^{*}/\left(\Gamma_{1}-i\delta \right)$, $\Pi_{1}=2\left(gf_{0}-i\Omega_{c}\right)-i\omega_{m}\beta S_{0}\eta$, $\Pi_{2}=i\left(\Delta_{c}-\delta +\omega_{m}\beta Q_{0}\right)+\Gamma_{2}-gw_{0}\Sigma_{1}-\Lambda_{2}\Pi_{1}$, $\Pi_{3}=2\left(gf_{0}-i\Omega_{c}\right)-i\omega_{m}\beta S_{0}\eta^{*}$, $\Pi_{4}=i\left(\Delta_{c}+\delta +\omega_{m}\beta Q_{0}\right)+\Gamma_{2}-gw_{0}\Sigma_{2}-\Lambda_{3}\Pi_{3}$. The imaginary and real parts of $\chi^{\left(1\right)}\left(\omega_{pr}\right)$ indicate absorption and dissipation, respectively.

### 2.4. Group Velocity Index

As the light group velocity as [35,36] $\upsilon_{g}=c/\left[n+\omega_{s}\left(dn/d\omega_{s}\right)\right]$ where $n\approx 1+2\pi \chi_{eff}^{\left(1\right)}$, then we obtain:

(19) $$c/\upsilon_{g}=1+2\pi Re{\left[\chi_{eff}^{\left(1\right)}\left(\omega_{s}\right)\right]}_{\omega_{s}=\omega_{ex}}+2\pi \omega_{s}Re{\left(d\chi_{eff}^{\left(1\right)}/d\omega_{s}\right)}_{\omega_{s}=\omega_{ex}}.$$

Obviously, when $Re{\left[\chi_{eff}^{\left(1\right)}\left(\omega_{s}\right)\right]}_{\omega_{s}=\omega_{ex}}=0$, the dispersion is steeply positive or negative and the group velocity is significantly reduced or increased. We further define the group velocity index $n_{g}$ as:

(20) $$n_{g}=\frac{c}{\upsilon_{g}}-1=\frac{c-\upsilon_{g}}{\upsilon_{g}}=\frac{2\pi \omega_{ex}\rho \mu^{2}}{\hbar \Gamma_{2}}Re{\left(\frac{d\chi_{eff}^{\left(1\right)}}{d\omega_{s}}\right)}_{\omega_{s}=\omega_{ex}}=\Gamma_{2}\Sigma Re{\left(\frac{d\chi_{eff}^{\left(1\right)}}{d\omega_{s}}\right)}_{\omega_{s}=\omega_{ex}},$$

where $\Sigma =2\pi \omega_{ex}\rho \mu^{2}/\hbar \Gamma_{2}^{2}$. One can observe the slow light if $n_{g}>0$ and the superluminal light when $n_{g}<0$ [37]. For the parameters of the hybrid QD–NR system [28], the exciton relaxation rate (the exciton dephasing rate $\Gamma_{2}$) $\Gamma_{1}=0.3$ GHz ($\Gamma_{2}=0.15$ GHz). The parameters NR are $(\omega_{n}$, *M*, $Q_{f})=(1.2$ GHz, $5.3\times 10^{-18}$ kg, $3\times 10^{4})$, where $\omega_{m}$, *M*, and $Q_{f}$ are the resonator frequency, the effective mass, and the quality factor of the NR, respectively. The decay rate of the NR is $\gamma_{m}=$$\omega_{m}/Q_{f}=40$ kHz, and the coupling strength is β=0.06. The parameters of MFs [3,4,5,6,24], the QD–MFs coupling strength g=0.1 GHz, the decay rate of the MFs $\kappa_{M}=0.1$ MHz, $\Gamma_{1}=0.3$ GHz, $\Gamma_{2}=0.15$ GHz, and $\Omega_{p}^{2}=0.1($GHz${)}^{2}$.

## 3. Numerical Results and Discussion

**Figure 2:**
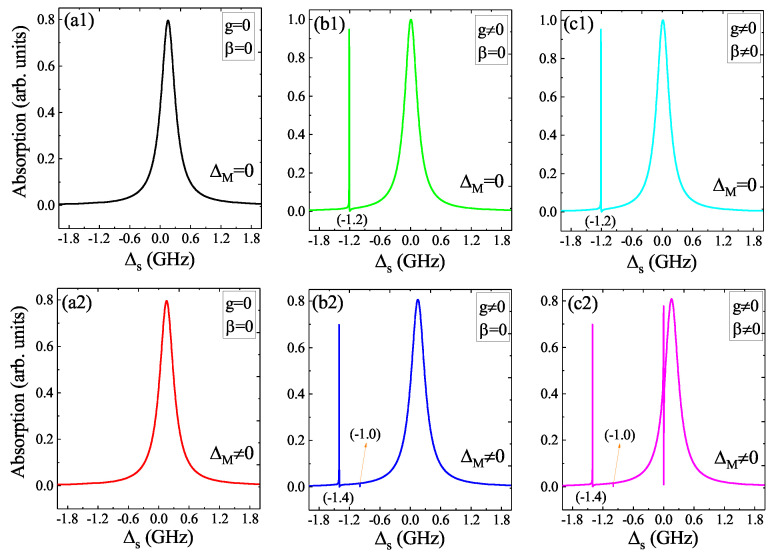
(**a1**,**a2**) The absorption spectra versus the probe–exciton detuning $\Delta_{s}$ for the parameters of g=0, β=0, $\Delta_{c}=\omega_{m}$ under the condition of $\Delta_{M}=0$ and $\Delta_{M}\neq 0$, respectively. (**b1**,**b2**) The absorption spectra versus $\Delta_{s}$ for the parameters of g=0.1 GHz, β=0, $\Delta_{c}=\omega_{m}$ under the condition of $\Delta_{M}=0$ and $\Delta_{M}\neq 0$, respectively. (**c1**,**c2**) The absorption spectra versus $\Delta_{s}$ for the parameters of g=0.1 GHz, β=0.06, $\Delta_{c}=\omega_{m}$ under the condition of $\Delta_{M}=0$ and $\Delta_{M}\neq 0$, respectively.

There are two kinds of coupling in the hybrid system, i.e., the QD–MF coupling and the QD–NR coupling, as shown in Figure 1, and in the QD–MF coupling regime, $\Delta_{M}=0$ or $\Delta_{M}\neq 0$, i.e., the uncoupled Majorana edge state or coupled Majorana edge state will also influence the coherent optical properties, so it is necessary to investigate the coherent optical spectra under different parameters and coupling regimes. Figure 2 shows the absorption spectra of the QD as a function of probe–exciton detuning $\Delta_{s}=\omega_{s}-\omega_{ex}$ at the red detuning ($\Delta_{c}=\omega_{m}$) for different parameters and coupling regimes. In Figure 2(a1,a2), we give the absorption spectra under $\Delta_{M}=0$ and $\Delta_{M}\neq 0$, respectively, for the parameters of g=0, β=0, and $\Delta_{c}=\omega_{m}$, i.e., there are no QD–MF coupling (g=0) and QD–NR coupling (β=0), and only the QD is driven by a pump laser and a probe laser, then the absorption spectra show a Lorentz peak. In Figure 2(b1,b2), we consider the QD–MF coupling (g≠0) without considering the QD–NR coupling (β=0). In the uncoupled Majorana edge state ($\Delta_{M}=0$), the absorption spectrum not only shows a Lorentz peak around $\Delta_{s}=0$, but also presents a sideband peak at $\Delta_{s}=-\omega_{m}$ ($\omega_{m}=1.2$ GHz), as shown in Figure 2(b1). In the coupled Majorana edge state ($\Delta_{M}\neq 0$, such as $\Delta_{M}=-0.2$ GHz), the absorption spectrum also displays a Lorentz peak around $\Delta_{s}=0$; however, the sideband peak at $\Delta_{s}=-\omega_{m}$ in Figure 2(b1) is split into two peaks located at $-\omega_{m}+\Delta_{M}$ (−1.4 GHz) and $-\omega_{m}-\Delta_{M}$ (−1.0 GHz), respectively, as shown in Figure 2(b2). In Figure 2(c1,c2), we not only consider the QD–MF coupling (g≠0), but also consider the QD–NR coupling (β≠0). In the case of $\Delta_{M}=0$, the absorption spectrum as shown in Figure 2(c1) shows the same result as shown in Figure 2(b1), i.e., in the uncoupled Majorana edge state ($\Delta_{M}=0$), the role of the QD–NR coupling (β≠0) is feeble and can be neglected. However, in the coupled Majorana edge state ($\Delta_{M}=-0.2$ GHz), if the QD–MF coupling (g≠0) and the QD–NR coupling (β≠0) simultaneously exist in the system, as shown in Figure 2(c2), we find that not only the absorption spectrum at $\Delta_{s}=0$ displays a Fano resonance (an asymmetric splitting), but also the sideband peak at $\Delta_{s}=-\omega_{m}$ shows a splitting located at $-\omega_{m}+\Delta_{M}$ and $-\omega_{m}-\Delta_{M}$, respectively. For the Fano resonance around $\Delta_{s}=0$ in the absorption spectrum, the physical origin of this result is due to the QD–MF coherent interaction, and a dressed state theory was introduced to interpret this physical phenomenon [38]. Therefore, the coupled Majorana edge state ($\Delta_{M}\neq 0$) combined with the QD–MF coupling and the QD–NR coupling will together influence the absorption spectrum, and then, it is necessary to research the role of the coupled Majorana edge state in the following.

**Figure 3:**
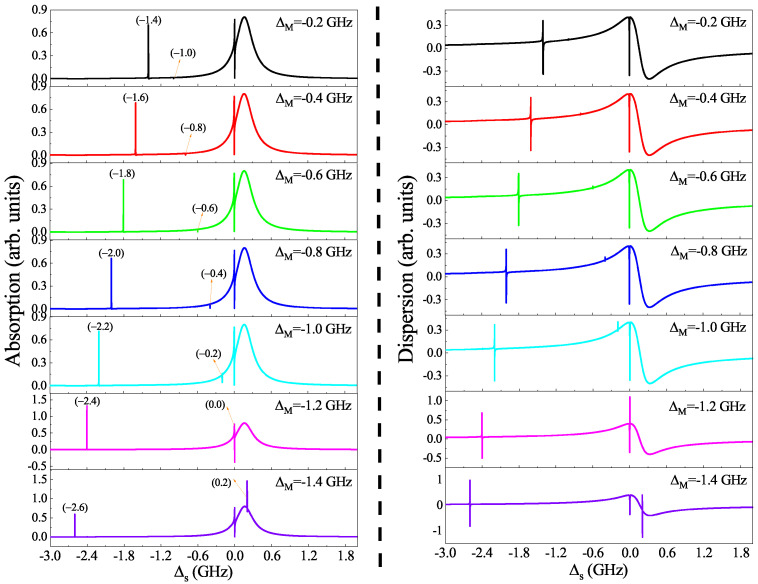
(**Left**) The absorption versus $\Delta_{s}$ for several different detuning $\Delta_{M}$. (**Right**) The dispersion versus $\Delta_{s}$ for several different detuning $\Delta_{M}$. The other parameters are g=0.1 GHz, β=0.06, $\Delta_{c}=\omega_{m}$, and $\Omega_{c}^{2}=0.1$ (GHz)${}^{2}$.

In Figure 3, we further present the absorption and dispersion profiles as a function of the probe detuning $\Delta_{s}$ at the parameters of g=0.1 GHz, β=0.06, and $\Delta_{c}=\omega_{m}$ for several different Majorana–pump detuning $\Delta_{M}$. The left parts show the absorption, and we can find that the Fano resonance induced by QD–MF coupling at $\Delta_{s}=0$ appear in the absorption spectra and the sideband peak induced by QD–NR coupling at $\Delta_{s}=-\omega_{m}$ is split into two peaks. With increasing the detuning $\Delta_{M}$ from $\Delta_{M}=-0.2$ GHz to $\Delta_{M}=-1.4$ GHz, in the two split peaks distributing in two sides of $\Delta_{s}=-\omega_{m}$, the left peak located at $-\omega_{m}+\Delta_{M}$ moves to the left, while the right peak located at $-\omega_{m}-\Delta_{M}$ moves to the right. The right parts plot the dispersion, and we show that the evolutionary process of the dispersion as a function of $\Delta_{s}$ with increasing detuning $\Delta_{M}$ from $\Delta_{M}=-0.2$ GHz to $\Delta_{M}=-1.4$ GHz, which combines the absorption, will induce the slow light effect because the dispersion manifests a positive steep slope around $\Delta_{s}=0$. The details of the absorption and dispersion around $\Delta_{s}=0$ are displayed in the following figures.

**Figure 4:**
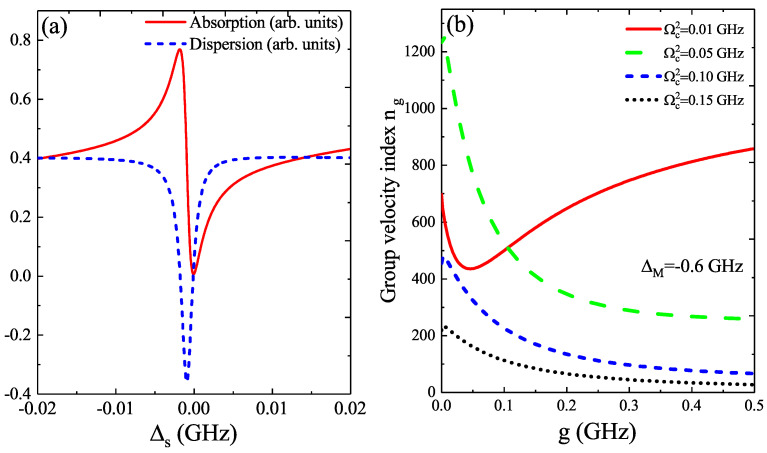
(**a**) The absorption and dispersion as a function of $\Delta_{s}$ at fixed $\Delta_{M}=-0.6$ GHz. (**b**) The group velocity index $n_{g}$ versus the QD–MF coupling *g* for four different Rabi frequencies $\Omega_{c}^{2}$ under the parameters of β=0.06, $\Delta_{M}=-0.6$ GHz, and $\Delta_{c}=\omega_{m}$.

Then, in Figure 4a, we take $\Delta_{M}=-0.6$ GHz as an example; the red curve shows the details of the absorption in Figure 3 around $\Delta_{s}=0$; the absorption spectrum presents Fano resonance, and the Fano shape is related to the detuning $\Delta_{M}$. The blue curve plots the details of the dispersion in Figure 3 around $\Delta_{s}=0$, and the dispersion is deep. However, no matter how the detuning $\Delta_{M}$ changes, there is still an invariant result, i.e., the dispersion shows a positive steep slope around $\Delta_{s}=0$. Therefore, we also investigated the Fano resonance-induced coherent optical propagation properties, as shown in Figure 4b. In Figure 4b, we plot the group velocity index $n_{g}$ versus the QD–MF coupling *g* for four different Rabi frequencies $\Omega_{c}^{2}$ under the parameters of β=0.06, $\Delta_{M}=-0.6$ GHz, and $\Delta_{c}=\omega_{m}$. We can see that the group velocity index $n_{g}$ behaves as the slow light effect, and the process of the evolution of the group velocity index $n_{g}$ is related to the QD–MF coupling *g*.

**Figure 5:**
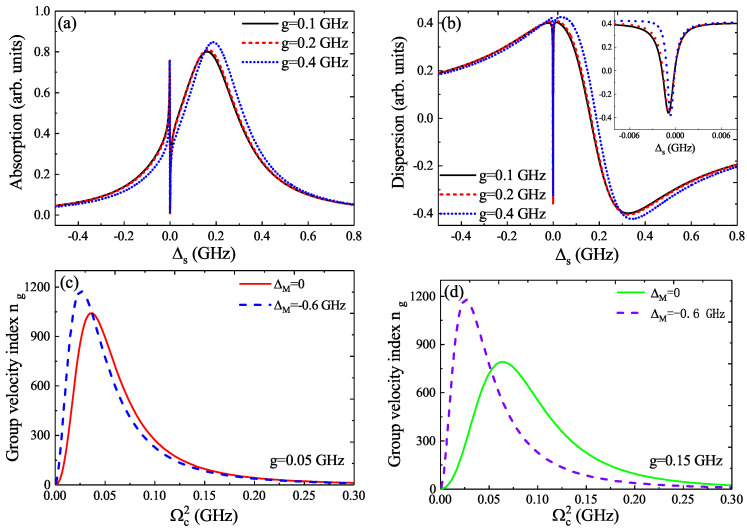
(**a**,**b**) The absorption and dispersion as a function of $\Delta_{s}$ for three different QD–MF coupling *g* under the parameters of β=0.06, $\Delta_{M}=-0.6$ GHz, $\Delta_{c}=\omega_{m}$, and $\Omega_{c}^{2}=0.1$ (GHz)${}^{2}$. (**c**,**d**) The group velocity index $n_{g}$ versus $\Omega_{c}^{2}$ for QD–MF coupling g=0.05 GHz and g=0.15 GHz, respectively.

Subsequently, in Figure 5a, we further investigate the absorption spectra as a function of $\Delta_{s}$ for three different QD–MF coupling *g* under the parameters of β=0.06, $\Delta_{M}=-0.6$ GHz, $\Delta_{c}=\omega_{m}$, and $\Omega_{c}^{2}=0.1$ (GHz)${}^{2}$, and we found that the splitting width of the absorption spectra is enhanced with increasing the QD–MF coupling *g*. Figure 5b displays the process of the evolution of the dispersion for three QD–MF coupling *g*, and the inset is the detail part around $\Delta_{s}=0$. We can obtain that the dispersion shows a positive steep slope around $\Delta_{s}=0$, which results in the slow light effect. Therefore, in Figure 5c, we plot the group velocity index $n_{g}$ as a function of the Rabi frequency $\Omega_{c}^{2}$ for $\Delta_{M}=0$ and $\Delta_{M}\neq 0$, respectively, at fixed QD–MF coupling g=0.05 GHz, and it is obvious that the group velocity index $n_{g}$ gradually increases and reaches a maximum, then decays to almost a zero value with increasing Rabi frequency $\Omega_{c}^{2}$, both at the coupled Majorana edge state ($\Delta_{M}\neq 0$) and uncoupled Majorana edge state ($\Delta_{M}=0$). If we consider a bigger QD–MF coupling, such as g=0.15 GHz, as shown in Figure 5d, we can find that the slow light effect (i.e., the process of the evolution of the group velocity index $n_{g}$) is more remarkable in the coupled Majorana edge state ($\Delta_{M}=-0.6$ GHz) than in $\Delta_{M}=0$.

**Figure 6:**
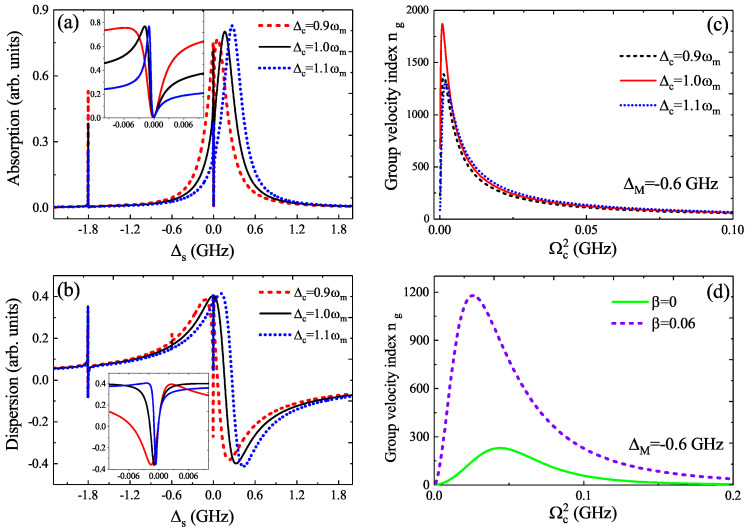
(**a**,**b**) The absorption and dispersion versus $\Delta_{s}$ for three $\Delta_{c}$ under the parameters of β=0.06, g=0.1 GHz, $\Delta_{M}=-0.6$ GHz, and $\Omega_{c}^{2}=0.1$ (GHz)${}^{2}$; the insets are their details. (**c**) $n_{g}$ as a function of $\Omega_{c}^{2}$ for three $\Delta_{c}$ at $\Delta_{M}=-0.6$ GHz. (**d**) $n_{g}$ versus $\Omega_{c}^{2}$ in the condition of β=0 and β≠0, respectively.

Except the QD–MF coupling *g*, the pump–exciton detuning $\Delta_{c}$ and the QD–NR coupling β can also affect the slow light effect. In Figure 6a, we give the absorption spectra as a function of $\Delta_{s}$ for three different pump–exciton detuning $\Delta_{c}$ under the parameters of β=0.06, g=0.1 GHz, $\Delta_{M}=-0.6$ GHz, and $\Omega_{c}^{2}=0.1$ (GHz)${}^{2}$, and it is clear that the absorption spectra show the mode-splitting behavior around $\Delta_{s}=0$ and that a transparent window (i.e., the zero absorption depth, as shown in the inset in Figure 6a) also appears in the absorption spectra. Figure 6b displays the dispersion for three different $\Delta_{c}$, and the dispersion has a positive steep slope around $\Delta_{s}=0$, as shown in the inset in Figure 6b. Thus, in Figure 6c, we plot the group velocity index $n_{g}$ as a function of $\Omega_{c}^{2}$ for three $\Delta_{c}$ at $\Delta_{M}=-0.6$ GHz, and we can see that the slow light effect is more obvious in $\Delta_{c}=\omega_{m}$ than in $\Delta_{c}\neq \omega_{m}$. We also investigated the role of the NR in the hybrid system that influences the slow light. The previous work demonstrated that the NR will behave as a phonon cavity, enhancing the coherent optical spectrum. Then, in Figure 6d, we compare the case of β=0 (no QD–NR coupling) and β≠0 (the QD–NR coupling strength β=0.06). If there is no QD–NR coupling, the slow light effect induced by MFs is demonstrated in a hybrid semiconductor/superconductor ring device [24], and it is similar to the result of the green curve in Figure 6d. However, if the QD–NR coupling is taken into consideration, such as the purple curve in Figure 6d, we can find that the group velocity index $n_{g}$ manifesting the slow light is enhanced significantly compared with the condition of no QD–NR coupling. Finally, by controlling the detuning regimes and the coupling regimes, the slow light can be reached in the hybrid system.

## 4. Conclusions

In conclusion, we demonstrated the coherent optical propagation properties in a hybrid device, which includes a QD driven by a pump field and a probe field implanted into an NR coupled to nearby MFs in superconducting iron (Fe) chains, and we investigated the absorption spectra of the probe field under different detuning regimes (such as pump–exciton detuning $\Delta_{c}$ and Majorana–pump detuning $\Delta_{M}$) and different coupling regimes (such as QD–MF coupling *g* and QD–NR coupling β). When the QD couples to nearby MFs, a Fano resonance can be obtained in the absorption spectra, which accompany the rapid phase dispersion inducing the slow light effect. The results showed that the group velocity index can be controlled by the QD–MF coupling, which can reach the slow light effect. Moreover, the NR was considered, which behaved as a phononic cavity, leading to an enhanced slow light effect.

## Data Availability

Not applicable.
